# Protein interaction mapping interpretation of none alcoholic fatty liver disease model of rats after fat diet feeding 

**Published:** 2017

**Authors:** Hamed Abdollahi, Mona Zamanian Azodi, Behzad Hatami

**Affiliations:** 1 *Proteomics Research Center, Faculty of Paramedical Sciences, Shahid Beheshti University of Medical Sciences, Tehran, Iran *; 2 *Gastroenterology and Liver Diseases Research Center, Research Institute for Gastroenterology and Liver Diseases, Shahid Beheshti University of Medical Sciences, Tehran, Iran*; 3 *Basic and Molecular Epidemiology of Gastrointestinal Disorders Research Center, Research Institute for Gastroenterology and Liver Diseases, Shahid Beheshti University of Medical Sciences, Tehran, Iran*

**Keywords:** Protein-protein interaction network analysis, Rat Model, None alcoholic fatty liver disease, Gene expression profile, Centrality analysis, Gene ontology

## Abstract

**Aim:**

This study investigates the effect of fat diet on gene expression profile in rat liver via protein-protein interaction mapping analysis.

**Background:**

Nonalcoholic fatty liver disease (NAFLD) is a prevalent condition of liver in the world. This progressive metabolic disease is representative with fat accumulation in the patients’ liver that can led to advance stages, namely, cirrhosis and eventually cancer.

**Methods:**

Differentially expressed genes of NAFLD rat liver after 2, 4 and 6 weeks fat diet feeding were analyzed via GEO2R and protein-protein interaction network by Cytoscape v3.6.0. and the related plug-ins. The important genes were assigned based on degree and betweenness centrality analysis and enriched using ClueGO+CluePedia Plug-in.

**Results:**

GAPDH, PRDM10, TP53, AKT1, INS, ALB, SRC, MAPK1, ACLY, ACACA, DECR1, ACACB, MBOAT4, TNF, EHHADH and JUN genes were introduced as key genes related to the fat diet fed NAFLD rats. Fatty acid biosynthesis and four other terms were introduced as the main related ones to the essential genes.

**Conclusion:**

The introduced critical genes and the related terms may describe NAFLD molecular condition and its progression to the other severe metabolic diseases. Moreover, these potential biomarkers may be monitored for diagnosis and treatment approaches after validation investigations.

## Introduction

 About one-third of the world population are affected by Nonalcoholic fatty liver disease (NAFLD) (1). The disorder manifests by excessive accumulation of fat in the liver tissue (2). Many metabolic disease are also correlated with NAFLD knowing as obesity, hyper insulinaemia, type 2 diabetes, insulin resistance, hyper triglyceridaemia and hypertension (1). On the other hand, this metabolic syndrome has a different stages that can trigger from simple liver condition to the severe diseases, in which, initiates from steatosis to non-alcoholic steatohepatitis, hepatocellular injury, fibrosis, and frequently lead to cirrhosis (3). What is more, it has also been known as a precursor of the ultimate stage of the liver which is hepatocellular cancer (4). In this light, monitoring this disease based on molecular concept can provide more information about disease nature and molecular changes during its differentiations. Gene expression profile plus PPI network analysis is one of the novel ways to reach this goal, which has been in a great attention recently (5-8). In a PPI approach, genes that are associated to a condition such as disease are interacted in a scale free network and the topological parameters of the constructed network analyzed for screening the studied genes (8). Topological criteria such as degree, betweenness centrality, closeness centrality and stress are used frequently for analysis of network of various diseases. Degree refers to the number of connections between one node and the other nodes of a network. The introduced crucial nodes in the format of a related panel can be used in biomarker discovery (9). Biomarkers are the biomolecules including proteins, metabolites, genes and the other reagents, which are specific and sensitive for a disease. Biomarker discovery is attracted more attention in the field of medicine (10, 11). Biomarkers are precise diagnostic tools and can be applied in follow up of patients (12). There are several documents about liver and its diseases via system biology particularly PPI network analysis, which provided new prospective to disease (13-16). The other important concept is gene ontology that analysis related biological processes, molecular functions, cellular components, and chemical pathways to the certain genes (17, 18). The aim of this research is to determine effect of fat diet feeding on protein interactome of NAFLD rat via studying gene expression profile of different stages that consequently may be useful in human molecular investigations. 

## Methods

Protein-protein interaction network analysis of significantly changed genes in expression is conducted in this study. The data is from the Gene Expression Omnibus (GEO)(19) in which the Platform (GPL1355) Series GSE73500, using Rat Genome 230 2.0 microarray (Rattus norvegicus), last update on Jul 31, 2017 is studied. This data is published as an article”Correlation Analysis Between Gene Expression Profile of Rat Liver Tissues and High-Fat Emulsion-Induced Nonalcoholic Fatty Liver” conducted by C Xu, et al. in 2011(20). The dataset consists of liver tissue samples of 12 rats of 12 weeks old from 4 stages, namely different time courses including normal liver tissue (0h) and after feeding with fatty food in 3 stages designed as 2 weeks, 4 weeks, and 6 weeks. As it is clear, each stage consists of 3 samples with accession IDs. 

**Figure 1:**
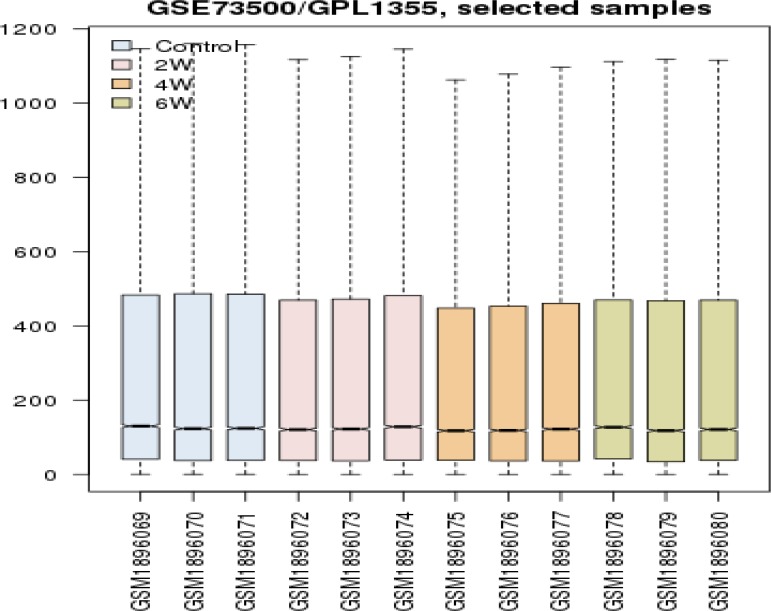
Comparison of expression amounts of the defined groups: three controls (blue color) and after 2 weeks feeding (pink color), after 4 weeks feeding (orange color), and after 6 weeks feeding (green color) are represented. The box-plot analysis was obtained using R statistical software. The x-axis and y-axis indicate the range of expression values and biological replications for groups, respectively. The comparison shows that the values are median-centered and consequently, the groups are comparable in terms of expression and further investigations are possible

**Table 1:** The central nodes (hub-bottleneck genes) related to PPI network of the two weeks feeding rats model of NAFLD with high fat diet in comparison with control group are presented

Row	Display name	Description	Degree	BC
1	GAPDH	Glyceraldehyde-3-phosphate dehydrogenase	125	0.03
2	PRDM10	PR domain containing 10	121	0.03
3	TP53	tumor protein p53	114	0.06
4	AKT1	v-akt murine thymoma viral oncogene homolog 1	113	0.02
5	INS	Insulin	106	0.03
6	ALB	Albumin	105	0.02
7	SRC	v-src sarcoma (Schmidt-Ruppin A-2) viral oncogene homolog (avian)	104	0.03
8	MAPK1	Mitogen-activated protein kinase 1	102	0.02

**Table 2:** The key nodes (hub-bottleneck genes) of PPI network of the four weeks feeding rats’ model of NAFLD with high fat diet in comparison with control group are shown

Display name	Description	Degree	BC
PRDM10	PR domain containing 10	86	0.08
ACLY	ATP citrate lyase	83	0.04
INS	Insulin	80	0.06
ACACA	Acetyl-CoA carboxylase alpha	79	0.03
DECR1	2,4-dienoyl CoA reductase 1, mitochondrial	79	0.03
GAPDH	Glyceraldehyde-3-phosphate dehydrogenase	78	0.03
ACACB	Acetyl-CoA carboxylase beta	75	0.03
ALB	Albumin	75	0.03
EHHADH	Enoyl-CoA, hydratase/3-hydroxyacyl CoA dehydrogenase	66	0.02

**Table 3:** The crucial nodes (hub-bottleneck genes) related to PPI network of the six weeks feeding rats model of NAFLD with high fat diet in comparison with control group are presented

Display Name	Description	Degree	BC
PRDM10	PR domain containing 10	104	0.04
GAPDH	Glyceraldehyde-3-phosphate dehydrogenase	101	0.03
ALB	Albumin	99	0.03
INS	Insulin	98	0.03
TP53	Tumor protein p53	95	0.05
DECR1	2,4-dienoyl CoA reductase 1, mitochondrial	92	0.04
MBOAT4	Membrane bound O-acyltransferase domain containing 4	91	0.02
TNF	Tumor necrosis factor	88	0.03
JUN	Jun proto-oncogene	87	0.02

**Table 4:** The central nodes (hub-bottleneck genes) related to PPI network of the feeding rats model of NAFLD with high fat diet in comparison with control group are presented. 2W, 4W and 6W are corresponded to two, four and six weeks feeding time courses respectively

**2W**	**4W**	**6W**
GAPDH	PRDM10	PRDM10
PRDM10	ACLY	GAPDH
TP53	INS	ALB
AKT1	ACACA	INS
INS	DECR1	TP53
ALB	GAPDH	DECR1
SRC	ACACB	MBOAT4
MAPK1	ALB	TNF
-	EHHADH	JUN

**Figure 2.**
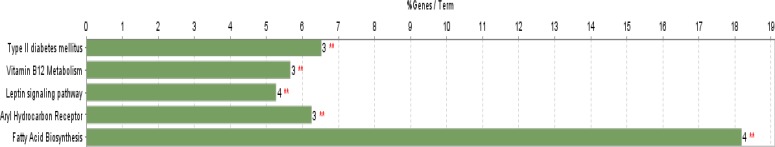
Gene ontology enrichment of relate 16 key genes to the PPI network of NAFLD rat after fat diet feeding. At least presence of three genes and 5% attribution for each term was considered. P-valve was less than 0.01

GEO2R, online GEO engine normalized the data and identified significantly altered genes in expression through assigning significance of (*p*≤ 0.05) along with the log transformation of fold change as well as considering the correction test of Benjamini & Hochberg (False discovery rate). Thousands of genes with other relevant information are detectable via GEO2R, however, here only the genes among the top 250 ranked significant ones (p≤ 0.05) with the fold change cutoff of ≥ 2 were chosen for further analysis. Accordingly, the R script provided by GEO2R was applied in the R Studio Software environment for conducting this evaluation as well, using GEO query and limma R packages from the Bioconductor project (21). Among these genes, those with gene name were searched against Cytoscape platform as the elements of interactions. Cytoscape in conjugation with String db (22, 23), constructed three different networks from comparison of different stages of normal/2w, normal/ 4w, and normal/6w versus control group. These networks were compared in terms of centrality features that were provided by the application of NetworkAnalyzer (24), as the well-integrated algorithm for computing topological parameters in Cytoscape. The well-known centrality attributes: degree and betweenness centrality were considered in this study to examine the potential effective agents that their removal causes perturbation of network structure (24). If degree value of a node was above Mean +2SD, the node was selected as hub-gene. The top 5% of the nodes based on betweenness value were introduced as bottleneck-genes (25, 26). The hub-nodes which were bottleneck determined as key genes (hub-bottleneck genes) (27). As the central nodes were assigned for our networks, the enrichment analysis was followed this step for the related biological processes applying ClueGO+CluePedia analysis (28). The CluePedia, in association of ClueGO, suggests more information to the related analysis by assigning some extended data (29). 

## Results

For logical comparison of the defined groups, expression values were investigated via box-plot analysis. The findings (see figure 1) show that the values are median-centered and consequently, the groups are comparable in terms of expression and further investigations is possible. Since organized genes in the integrated network can provide useful information about the roles of the involved genes in the disease, the related PPI networks for the three groups were constructed and analyzed. 

**Figure 3.**
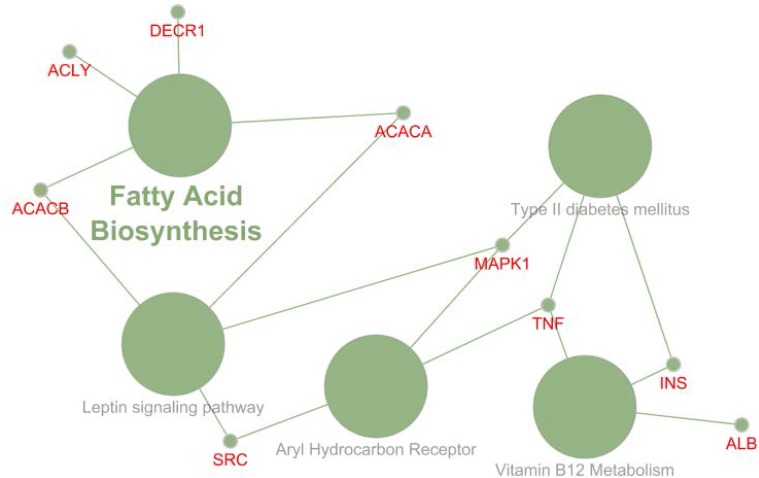
Gene ontology enrichment of relate 16 key genes to the PPI network of NAFLD rat after fat diet feeding is presented. The pathway and related genes are shown. At least presence of three genes and 5% attribution for each term was considered. P-valve was less than 0.01

The scale free networks (data are not shown) were used as suitable tools for gene screening. The key genes based on degree values and amounts of betweenness centralities determined for each networks and results are tabulated in the tables 1-3. The introduced nodes in these tables are highlighted as hub-bottleneck genes. For better comparison between the studied groups, content of tables 1-3 was summarized in table 4. In this table, the central genes can be compared based on feeding time courses. Overall combination of the key genes related to the three studied groups including GAPDH, PRDM10, TP53, AKT1, INS, ALB, SRC, MAPK1, ACLY, ACACA, DECR1, ACACB, MBOAT4, TNF, EHHADH and JUN genes were determined. This combination was obtained from content of table 4 by selection the genes with one-time repetition. Since the roles of the central genes in disease are crucial factors for clear interpretation of finding, Gene ontology enrichment for the 16 mentioned key genes was done by ClueGO software and the finding were presented in the figures 2,3. 

The statistical analysis for gene ontology are as follow: Kappa statistic is used for analysis how much the biological terms are grouped together as clusters. Kappa Score: 0.4, Min level of ontology: 3 Max level of ontology: 8, Number of min gene per term: 3 and 3, Percentage of min gene per term: 5, the correction method: Bonferroni step down, Enrichment/depletion test for the terms: 2-sided enrichment/depletion based on hypergeometric method.

## Discussion

Fatty liver disease is the most prevalent type of liver disorder worldly that can lead to other kinds of serious disorders such as cirrhosis, diabetes, heart attack and stroke (30). Molecular study can be helpful for better recognition of the disease root and associated mechanisms that promote the condition (26). In the present study, the key genes related to the feeding style including fat diet of rat model of NAFLD are investigated via PPI network analysis. As depicted in the tables 1 and 4, a key gene profile including eight central genes (GAPDH, PRDM10, TP53, AKT1, INS, ALB, SRC and MAPK1) is highlighted in the PPI network of the rat model of NAFLD after two weeks feeding with high fat diet. As it is shown in tables 2, 3 and 4, the key genes profile as a dependent variable of time feeding is changed after four and six weeks of feeding. As it is shown in the table 4, there are five common genes between the three studied groups. Due to the fact that TP53 is close to the cut off assigned in 4 w group, it is possible to consider it as a common gene. 

In the first step, combination of all of the key genes can be introduced as a panel including GAPDH, PRDM10, TP53, AKT1, INS, ALB, SRC, MAPK1, ACLY, ACACA, DECR1, ACACB, MBOAT4, TNF, EHHADH and JUN genes. This panel independent of feeding time is corresponded to the effect of fat diet on the NAFLD gene expression profile especially the key genes. Since there are five common genes for each group, it can be concluded that about 50-60% of the introduced genes for individual groups are common genes. In the second glance, it is clear that AKT1, SRC and MAPK1 are the specific crucial genes that refer to the early effect of fat feeding. Finally, MBOAT4, TNF and JUN are appeared in the last time course feeding. Moreover, the roles of the 16 key genes in NAFLD rats after feeding fat diet were investigated via gene ontology analysis. For achieving important pathways, the restricted investigation was applied to GO enrichment. The findings are shown in the figures 1 and 2. Nine genes among 16 genes were included in the analysis. Absence of MBOAT4, TNF and JUN, the special key genes of 6 w group and presence of ACACA, ACACB and ACLY, the distinctive key genes of 4 w group among 16 key genes indicates that the early time courses of feeding have considerable effect on the introduced profile. Membrane-bound *O*-acyltransferase 4 (MBOAT4) is a hydrophobic enzyme that esterifies long-chain fatty acids to the target proteins (31). This enzyme is involved in reduction of hepatic autophagy (32). It is reported that anti-TNF antibodies inhibit inflammation and improve NAFLD. TNF evokes hepatic inflammatory response in liver in the NAFLD patients (33). Investigation shows TNF induces c-JUN in liver regeneration response (34). Based on these finding, it can be concluded that MBOAT4, TNF and JUN are related to the advanced stage of fatty diet feeding course in the NAFLD rats. Fatty acid biosynthesis is the main biochemical pathway which is involved with four key genes (see figures 1, 2). This is one of the well-known and primary pathways in metabolism (35). Since fat diet feeding is accompanied by intake of extra amounts of fatty acids; it is predictable that this pathway be induced. Leptin signaling pathway is the other pathway which is correlated with four genes. Release of leptin from adipocytes plays an important role in body weight regulation. Several signal transduction proteins such as STAT3, SOCS3, PTP, IRS, AMPK and SH2B; are mediated in this pathway which right function of them is required (36). Therefore, alteration of body weight after fat diet feeding is not avoidable. About 70% of cases with type 2 diabetes mellitus are also dealing with NAFLD (37). The third pathway is concerned with three genes and describes this relationship. Role of aryl hydrocarbon receptor in toxicity and cancer is discussed in several documents (38). Negative correlation between vitamin B12 and NAFLD is reported (39). MAPK1 and TNF are the two genes which each of them were connected to the three terms. Relationship between MAPK1 and various cancer types is confirmed (40, 41). Role of TNF in cancer has attracted attention of researchers and there are several documents about its significant role (42, 43). Acetyl-coA carboxylase as a main genes in correlation with fatty acid biosynthesis has an essential role in fatty acid metabolism in the animal tissue and is a criterion for investigation on the epigenetic effects on fatty acid metabolism (44). Albumin as a housekeeping gene plays an important role in body hemostasis. Drug transfer, carrier of metabolites, hormones and several types of biomolecules in body are the well-known definition of albumin (45). This broad spectrum of functional roles of albumin in body implies strong expression change of albumin in correlation with most of diseases. Expression change of DECR1; the auxiliary enzyme in β-oxidation of fatty acids is reported in several cancers (46). Relationship between this enzyme and fatty acid metabolism is led to introduce DECR1 as a meat quality marker (47). Since inflammatory response in liver of NAFLD patients can progress to cirrhosis or even liver cancer and disease is strongly associated with two health problem including obesity and insulin resistance (48), The findings of this research are corresponded to the literature and represent the new insight of NAFLD. The findings are focused on metabolism of fatty acids and risk of the other diseases especially liver cancer. It is PPI network analysis power that highlights a few crucial genes among numerous of reported genes in correlation with a certain disease. 

Among query genes and their related ones, 16 genes were introduced as key elements in relationship with five essential pathways which are involved in the NAFLD rat in fat diet feeding course. Most of the enriched genes refer to four weeks fat diet feeding group which represent time course of feeding. It seems that the introduced genes and pathways play significant roles in pathology of NAFLD and its progress to the other diseases in particular cancer.
